# The significance of deformation mechanisms on the fracture behavior of phase reversion-induced nanostructured austenitic stainless steel

**DOI:** 10.1038/s41598-018-26352-1

**Published:** 2018-05-21

**Authors:** R. D. K. Misra, V. S. Y. Injeti, M. C. Somani

**Affiliations:** 1 Laboratory for Excellence in Advanced Steel Research, Department of Metallurgical, Materials and Biomedical Engineering, University of Texas at El Paso 500W, University Avenue El Paso, El Paso, TX 79968-0521 USA; 2 Center for Advanced Steel Research, The University of Oulu, P.O. Box 4200, 90014 Oulu, Finland

## Abstract

We describe here the relationship between grain structure, deformation mechanism and fracture characteristics in an austenitic stainless steel. This was accomplished using the novel concept of phase reversion that enabled a wide range of grain size from nanograined/ultrafine grained (NG/UFG) to coarse-grained (CG) regime to be obtained in a single material through change in temperature-time annealing sequence. In the NG/UFG structure, a marked increase in abundance of stacking faults (SFs) and twin density with strain was observed that led to a decrease in the average spacing between adjacent SFs, thus converting stacking faults into twins. Twinning in NG/UFG structure involved partial dislocations and their interaction with the grain boundaries, including SF overlapping and the coordinated nucleation of partial dislocations from the grain boundaries. The plastic zone in the NG/UFG structure resembled a network knitted by the intersecting twins and SFs. With SFE ~30 mJ/m^2^, the minimum stress for twin nucleation was ~250 MPa for the experiment steel and the corresponding optimal grain size (d_op_) wa ~120 nm. In contrast, in the CG structure, strain induced martensite formation was the deformation mechanism. The difference in the deformation mechanism led to a clear distinction in the fracture behavior from striated fracture in high strength-high ductility NG/UFG alloy to microvoid coalescence in the low strength-high ductility CG counterpart. The underlying reason for the change in fracture behavior was consistent with change in deformation mechanism from nanoscale twinning in NG/UFG alloy to strain-induced martensite in the CG alloy, which is related to change in the stability of austenite with grain size. An analysis of critical shear stress required to initiate twinning partial dislocations in comparison to that required to nucleate shear bands is presented. The appearance of striated fracture in the NG/UFG alloy suggests a quasi-static step wise crack growth process.

## Introduction

There is a strong interest to understand and develop advanced high strength steels, including austenitic stainless steels that are characterized by nano/ultrafine grains with high strength-high ductility combination as light-weight structural materials. Traditional austenitic stainless steels have low yield strength of 350–450 MPa. Grain refinement is a practical approach to enhance the yield strength of metallic materials^1,2^. Thermo-mechanically controlled processing (TMCP) is a widely used approach to refine the grain size. The combination of TMCP and microalloying elements led to ferrite grain size of less than 5 µm^3–6^. However, there is limit to which grain size can be refined by TMCP. On the other hand, severe plastic deformation such as equal channel angular processing, hot torsion, multiaxial forging provides an opportunity to obtain submicron or ultrafine grain structure in metals and alloys^7,8^.

**Figure 1:**
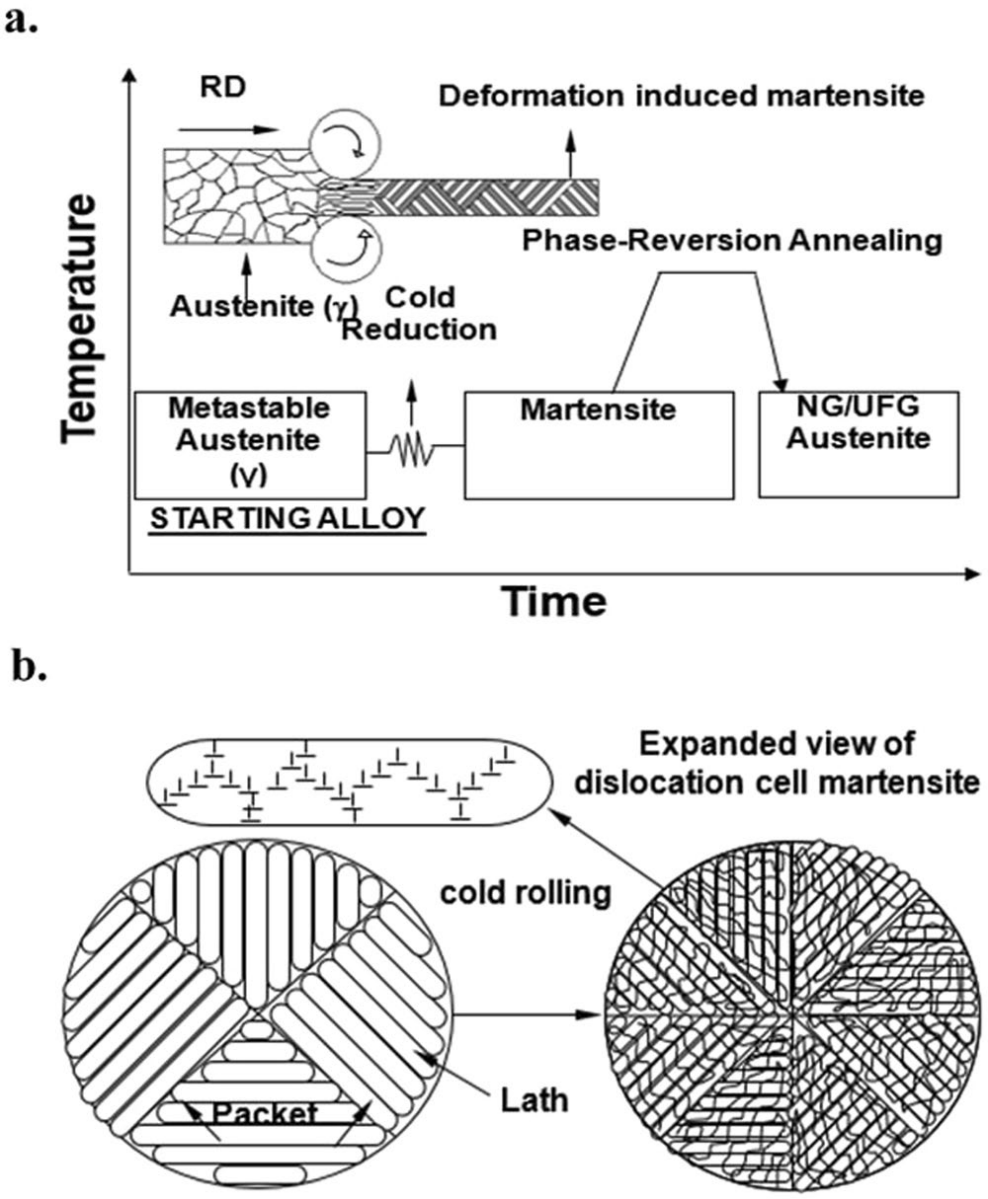
(**a**) A schematic representation of phase reversion concept to obtain NG/UFG structure^9–12^. Similar approach has been proven for microalloyed steels. (**b**) A schematic of refinement of packet and lath size for dislocation cell-type martensite during cold deformation (packet refers to a set of laths in a particular orientation).

We have recently developed an innovative concept of phase reversion (Fig. 1) to obtain nanograined/ultra-fine grained (NG/UFG) structure in austenitic stainless steels^9–12^. The concept involves cold deformation (~60–80%) of metastable (FCC) austenite (γ) to strain-induced body-centered cubic (BCC) martensite (α′), followed by phase-reversion annealing normally in the range 700–800^o^C for short durations, following which martensite reverts to austenite via diffusional or shear mechanism, depending on the chemical composition of the steel^9–12^. The unique aspect of the phase reversion concept is that a wide range of yield strength can be obtained in a single material depending on the grain size varying in a wide regime from nano-grained (NG) to coarse-grained (CG), by altering the degree of cold deformation and annealing temperature-time sequence.

It is now widely recognized that the deformation mechanisms in NG/UFG structure can be significantly different from those occurring in the CG structure. The higher strength of NG materials in relation to the CG counterpart has led to the suggestion that the primary mode of plastic deformation operating in ductile CG materials comprising mediation of the grouped activity of dislocations within the grains (e.g., dislocation pile-up and cells) is suppressed in the NG structure, where partial dislocation emission from grain boundaries may operate^13,14^. It is believed that the decrease in grain size and consequent increase in yield strength must be accompanied by a change in the deformation mechanism.

The present study focuses on the dependence of grain structure on the deformation behavior and consequent fracture mechanism. In this context, we have used the innovative concept of phase reversion to obtain NG/UFG to CG structure in a single material through change in the reversion annealing temperature-time sequence.

## Material and Experiment

The nominal chemical composition of the experimental austenitic stainless steel (in wt.%) was Fe-0.017C-0.52Si-1.3Mn-17.3Cr-6.5Ni-0.15Mo-0.15 N. Strips of stainless steel were initially subjected to severe cold rolling reduction of ~60% in an in-house laboratory rolling mill, followed by reversion annealing at 700–900 °C for 10–100 s in a Gleeble 1500 thermo-mechanical simulator. The objective of annealing at different temperature-time combination was to obtain different grain size from NG/UFG to CG structure. The experimental details are described in detail elsewhere^10,12,15–17^.

The progress in deformation processes during tensile straining as a function of strain was monitored via post-mortem analysis of the tensile deformed region within the gage length by transmission electron microscopy. 3 mm discs were punched and mechanically ground and electropolished in an electrolyte containing 10% perchloric acid in acetic acid at a temperature of 10 °C. Tensile samples tested until fracture were examined in a scanning electron microscope (SEM) to study the mode of fracture. The SEM micrographs of the fracture surface were processed using Image Pro software to clearly delineate the fracture morphology.

## Results

**Figure 2:**
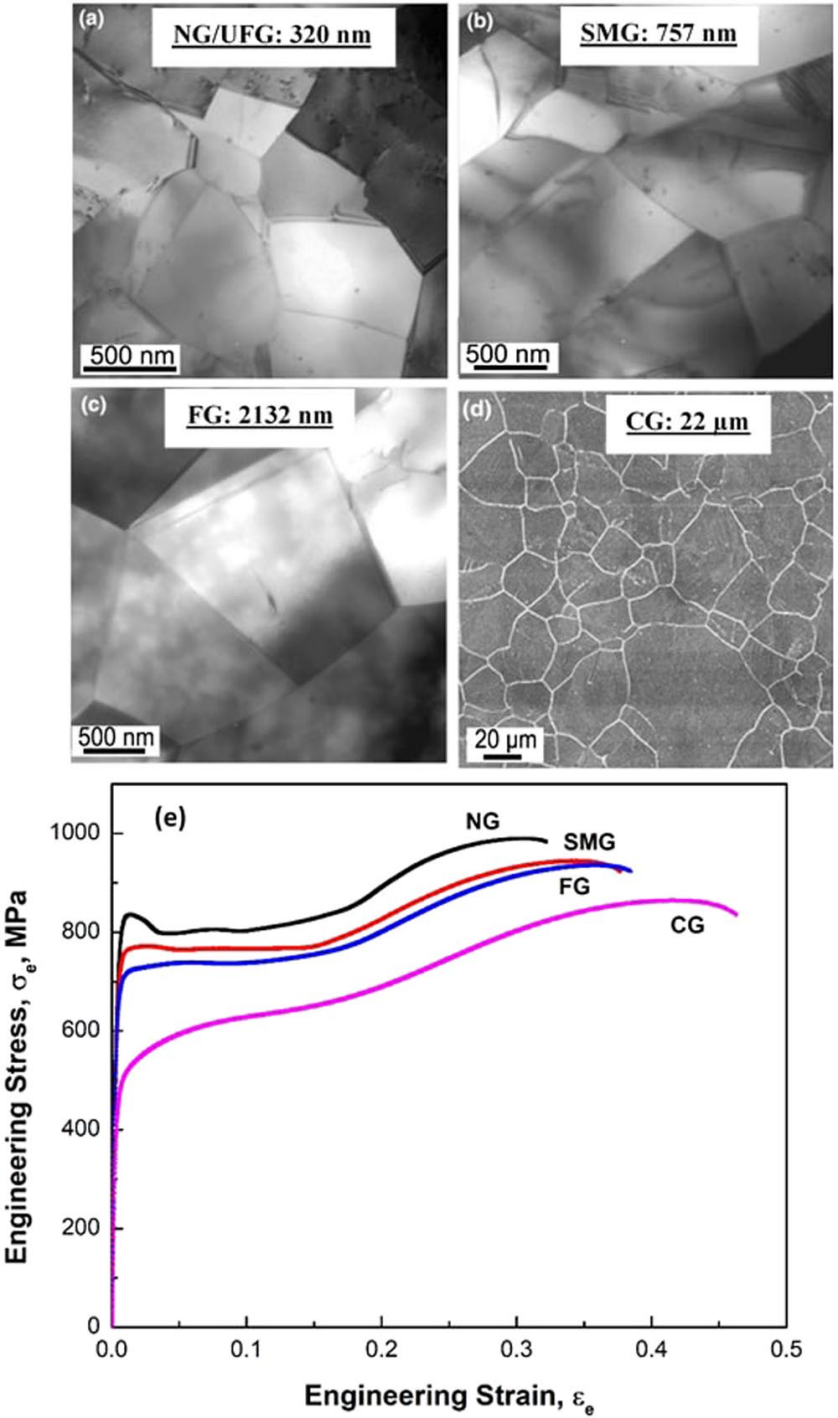
(**a**–**c**) TEM micrographs of phase reversion annealed 301LN type austenite stainless steel with varying grain size from nanograined/ultrafine-grained (NG/UFG) regime to fine-grained (FG) regime and (**d**) light micrographs of coarse-grained (CG) steel. The average weighted grain size ${\bar{d}}_{w}$ was determined from a number of micrographs and is indicated on each of the micrographs. NG/UFG: nanograined/ultrafine grained, SMG: sub-micron-grained, FG: fine-grained, and CG: coarse-grained steels^12^ (**e**) stress-strain plots for NG/UFG, SMG, FG and CG steels.

Figure 2 summarizes the grain structure of cold worked and phase reversion annealed austenitic stainless steel with different grain size, namely, nanograined/ultrafine-grained (NG/UFG), sub-micron grained (SMG), fine-grained (FG) and coarse-grained (CG). The mechanical properties for these grain structures are listed in Table 1. The weighted average grain size of NG/UFG, SMG, FG and CG was 320 ± 5 nm, 757 ± 10 nm, 2132 ± 21 nm and 22 ± 3 μm, respectively. The average number indicated on the micrographs is an average grain size determined from a large number of TEM micrographs and at least 3 specimens for each condition. The details of measurement approach are described in detail elsewhere^17^. Numerous high angle grain boundaries had misorientation between 57.5–62°. It may be noted that in spite of increase in yield strength with decrease in grain size to NG/UFG regime, the ductility (tensile elongation) continued to be high for all the steel samples.

**Table 1:** Tensile properties of phase reversion-induced Fe-17Cr-7Ni austenitic alloy with different grain size (data revised and adapted from ref.^16^).

	Weighted Average Grain Size	Average Yield Strength, MPa	% Average Elongation
NG/UFG	320 ± 5 nm	768	34
SMG	757 ± 10 nm	722	38
FG	2132 ± 21 nm	667	41
CG	22 ± 3 μm	350	40

### Interplay between grain structure and deformation behavior

**Figure 3:**
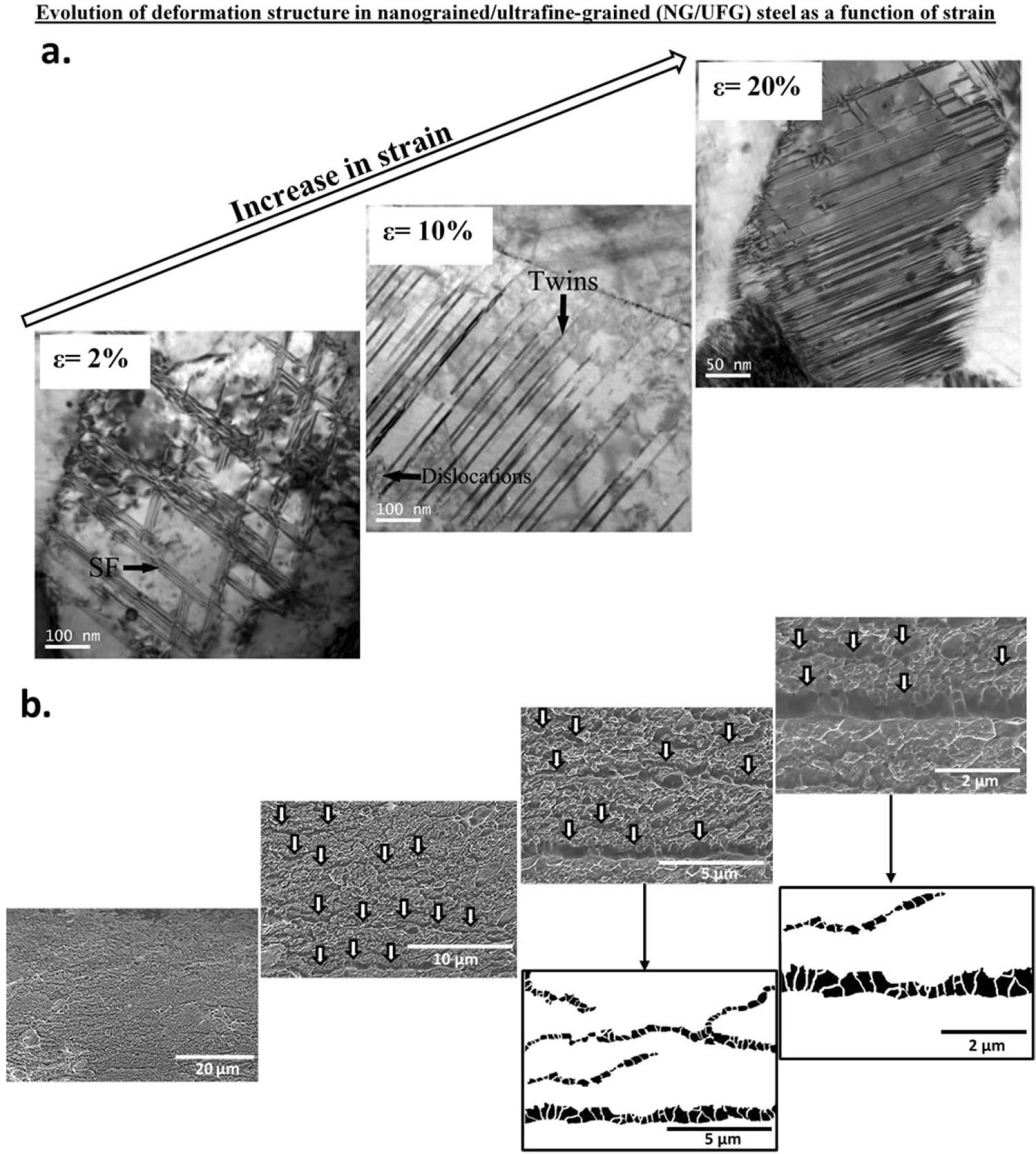
(**a**) TEM micrographs of NG/UFG structure illustrating evolution of deformation structure during tensile straining at engineering strain of 2%, 10% and 20% (partially adapted from ref.^16^), (**b**) SEM fractographs at different magnifications illustrating line-up of voids along the striations in NG/UFG steel. Also presented images processed fractographs.

**Figure 4:**
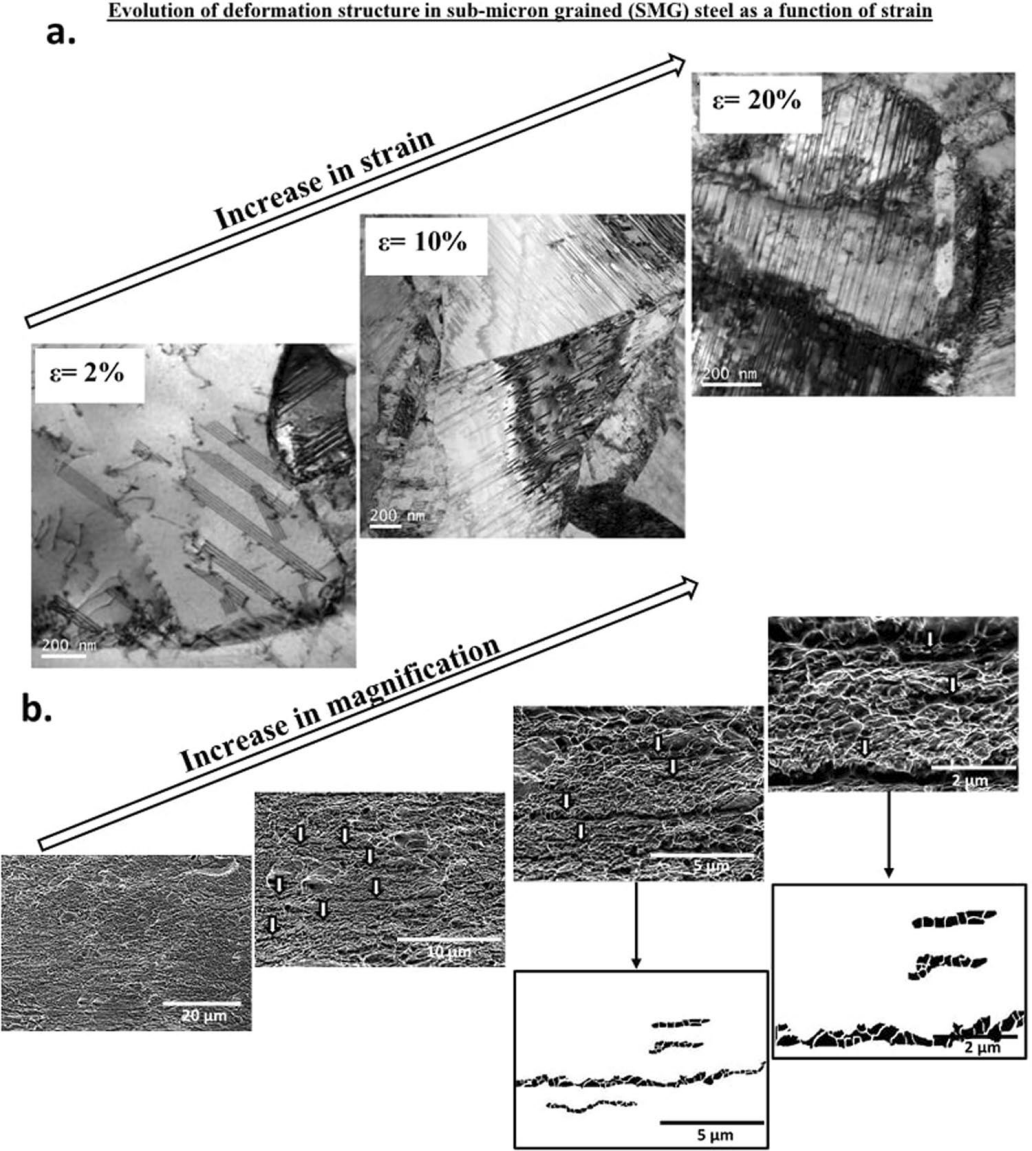
(**a**) TEM micrographs of SMG structure illustrating evolution of deformation structure during tensile strains at engineering strain of 2%, 10% and 20% (partially adapted from ref.^16^), (**b**) SEM fractographs at different magnifications illustrating line-up of voids along striations in SMG steel. Also presented are image processed fractographs.

**Figure 5:**
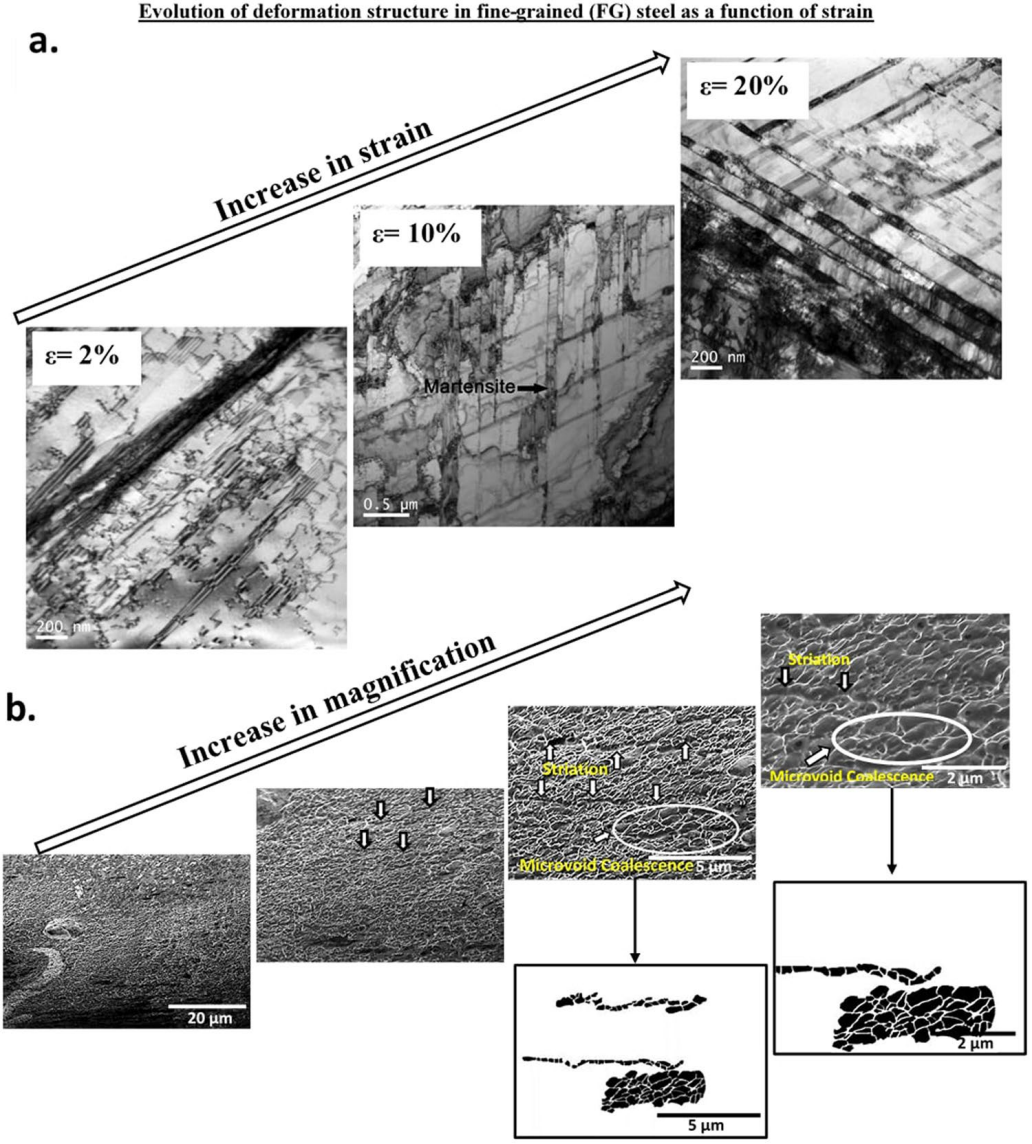
(**a**) TEM micrographs of FG structure illustrating tensile strain-induced deformation structure at engineering strain of 2%, 10% and 20% (partially adapted from ref.^16^), (**b**) SEM fractographs at different magnifications illustrating line of voids along the striations and microvoid coalescence type of fracture in FG steel.

**Figure 6:**
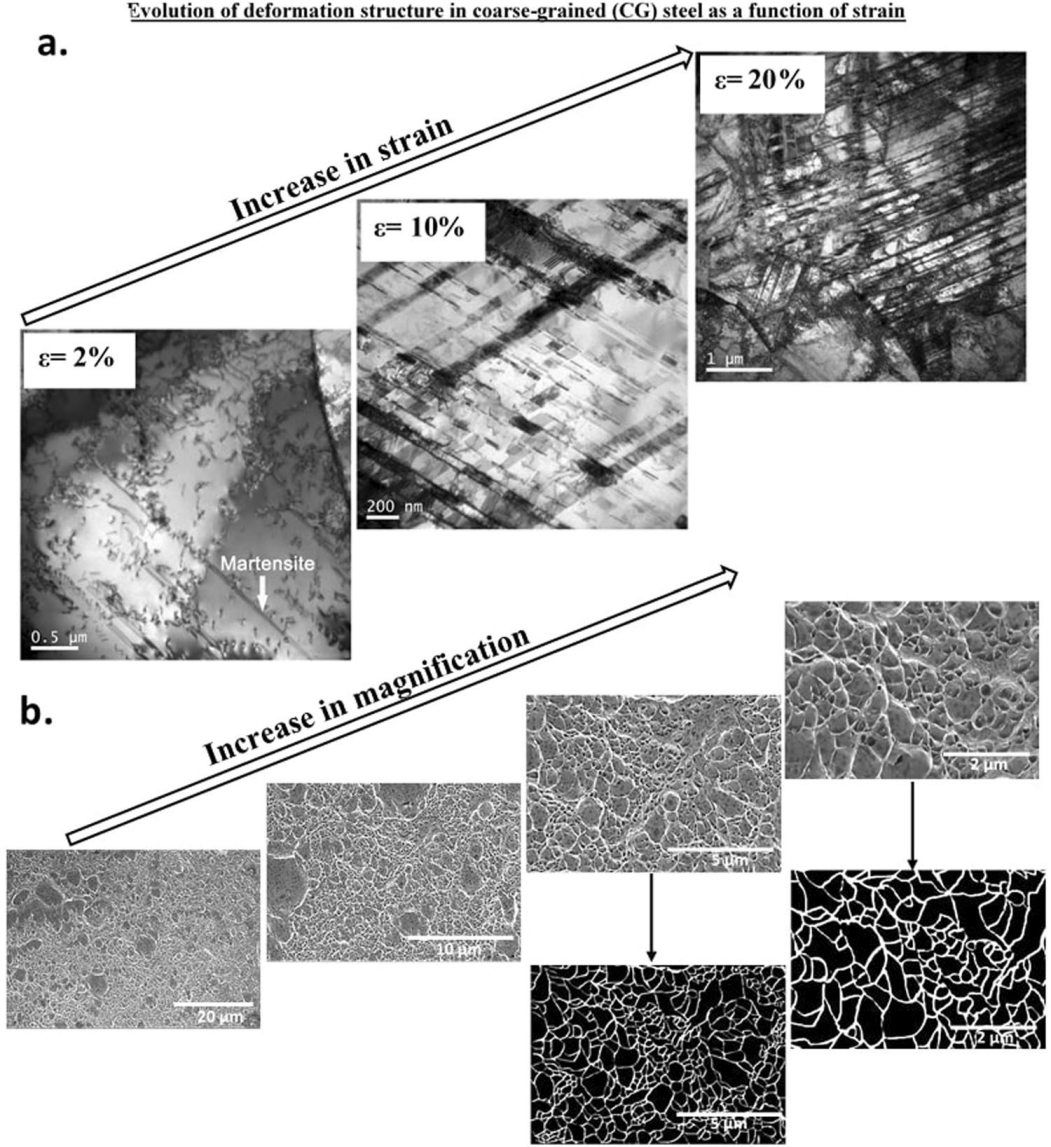
(**a**) TEM micrographs of CG structure illustrating tensile strain-induced deformation structure at engineering strain of 2%, 10% and 20% (partially adapted from ref.^16^), (**b**) SEM fractographs at different magnifications illustrating microvoid coalescence type of fracture in CG steel.

Transmission electron micrographs illustrating the evolution of deformation structure with increase in tensile strain, final fracture and processing of SEM fractographs by Image Pro for NG/UFG, SMG, FG and CG structures are presented in Figs 3–6. In NG/UFG austenitic stainless steel, there were a number of stacking faults (SF) during the early stages of plastic deformation, followed by increase in twin density with increased tensile strain. In SMG austenitic stainless steel, there was a similar increase in the number of nanoscale twins with increase in tensile strain. Twins nucleated in different directions and intersected one another at high strain.

The progress of deformation and microstructural evolution with tensile straining in FG austenitic stainless steel with weighted average grain size of ~2 μm (2132 nm) was intriguing. In addition to nanoscale twins, strain-induced α′-martensite at shear bands was also observed. The deformation mechanisms in the FG structure involved mechanical twinning and strain-induced martensite. Thus, in contrast to NG/UFG and SMG steels, in FG steel two different deformation mechanisms were operative, i.e. twinning and strain-induced martensite. In the CG structure, only strain-induced martensite was observed. Thus, we can conclude that there was a clear and distinct transition in the mechanism of deformation from NG/UFG to CG structure. This transition occurred when the weighted average grain size was ~2132 nm (i.e. FG structure).

It is appropriate to briefly discuss stacking faults and twins in the context of NG/UFG, SMG and to some extent in FG structure. These structures are typical of deformed low stacking fault energy (SFE) materials characterized by planar arrays of dislocations, due to the fact that the partials are widely separated and the cross-slip process of screw dislocations is difficult. One can clearly identify lots of SFs and twins present in the deformed area of the NG/UFG sample (Figs 3–5). The networks knitted by the intersecting SFs and twins were noted everywhere in NG/UFG and SMG steels. High densities of SFs can also be regarded as micro-twins with the thickness of only one atomic layer. These micro-twins and SFs were confined within the grain, and stopped in the grain interior with Shockley partial dislocations located at the tip of micro-twins and SFs (Figs 3–5). It is conceivable that these twins were heterogeneously nucleated at a grain boundary (GB) and grew into the grain interior via partial dislocation emission from the GB. It has been demonstrated that deformation twins can form by successive emission of Shockley partial dislocations from the same GB on adjacent (111) planes^18–21^, as also supported by molecular dynamic simulations^22^. The simulations revealed a high concentration of SF planes in the grains produced by single partials or by dissociated perfect dislocations. These SFs confined by the dense GB network of nanoscale grains were found to initiate deformation twinning, the underlying twinning mechanisms involve partial dislocations and their interaction with the GBs, including overlapping SF and the coordinated nucleation of partial dislocations from the GBs.

Dislocation activity is a precursor to twinning, and subsequently twinning proceeds as an energetically favorable rearrangement of partial dislocations (SFs). In other words, SFs always form before the occurrence of a twin. Meanwhile, the reason for the high density of SFs in the deformed samples is associated with the fact that smaller grains make it easier to emit partial dislocations than to emit perfect dislocations from GBs^22–28^. This makes partial dislocation emission from GBs a major deformation mechanism when the grain size is below a certain critical value, which is the case in the present study.

To date, it is well established that strain hardening and grain boundary (GB) hardening are the two major mechanisms to strengthen a metal, resulting from the impeded movement of dislocations by increasing density of obstacles-dislocations and GBs. The formation of stacking faults (SFs) as observed here, has recently come to play an important role in the understanding of superior mechanical properties of materials, particularly those with the face-centered cubic (fcc) and the close-packed hexagonal (hcp) structures. The improved properties include work hardening, recrystallization, creep, deformation texture, corrosion resistance and a number of others have been shown in general to be related to the presence of SFs^20^.

On the other hand, the high concentration of grain boundaries (GBs) in NG/UFG structure act as barrier to motion of dislocations and consequently enhances strength. However, GB strengthening mechanism alone provides limited contribution to macroscopic yield strength.

**Figure 7:**
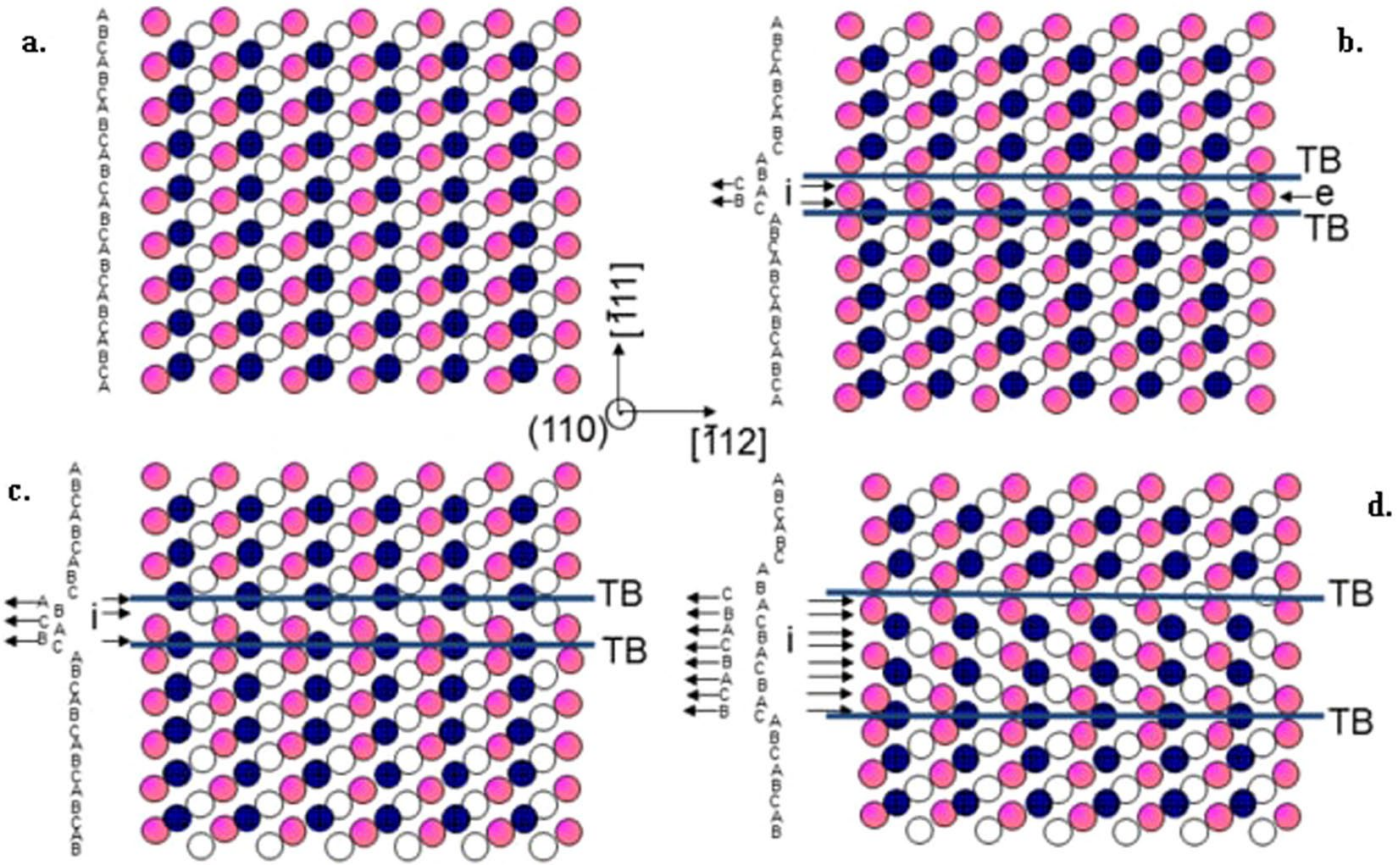
Schematic illustrations of stacking faults and micro-twin in an fcc lattice. (**a**) Perfect lattice with stacking sequence of ABC. (**b**) 2-layer intrinsic fault being equivalent to a single extrinsic fault. (**c**) A micro-twin with a thickness of 3-layer atoms clearly consisted of three overlapping intrinsic fault. (**d**) Unambiguously, the layer number of twin in coincidence with that of intrinsic faults on every (111) plane. : Atomic layer A, ○: atomic layer B, : atomic layer C on every (−11–1) plane; i: intrinsic fault, e: extrinsic fault, TB: twin boundary.

Given that the SFs and twins were consistently observed in the NG/UFG structure, it is pertinent to briefly revisit the understanding of the relationship between twin and SFs; the schematic illustrations are presented in Fig. 7. The fcc and hcp structures are closely related and, both being close-packed, differ essentially in the way in which the closest-packed planes are stacked together. The normal sequence of {111} planes in a face centered cubic (fcc) structure can be described as ABCABCABC using the usual A, B, C notation, as shown in Fig. 7a. The three typical stacking faults are illustrated by the characteristic stacking patterns in Fig. 7b–d. i.e., the intrinsic SF, extrinsic SF and A micro-twin with a thickness of *N* atomic layers resulted from *N* SFs on every (111) plane^18,19,28^. The key features can be described as follows:

(i) the intrinsic SF, corresponding to the removal of a close-packed layer of atoms (Fig. 7b). Moreover, one intrinsic SF can be considered as a micro-twin with one atomic layer. And the twin boundaries characterized by intermixed regions of hcp structure^18,19,28^.

(ii) The extrinsic SF, corresponding to the insertion of an extra close-packed layer of atoms, and 2-layer overlapping intrinsic SF is equivalent to a single extrinsic SF (Fig. 7b). Therefore, one extrinsic SF can be seen as a two-layer micro-twin and three overlapping intrinsic SFs form a 3-layer micro-twin with contacting hcp regions (Fig. 7c).

(iii) A micro-twin with a thickness of *N* atomic layers resulted from *N* SFs on every (111) plane (*N* = 8, Fig. 7d).

It is well demonstrated that twinning is an effective approach for improving strength and ductility for metals and alloys^29,30^ and enhance strain hardening^31^, which is consistent with our observations. SFs on basal planes are expected to provide similar effect on impeding dislocation movement as reported for nano-twinned electrodeposited Cu^32^. The high density of dislocations between SFs suggests that SFs are effective in blocking and accumulating dislocations (Fig. 3).

Because of the low SFE of austenitic stainless steel, the partial dislocations that constitute a glide dislocation are more widely separated, or extended, than in higher SF materials. Before a dislocation can move off its primary slip plane onto a cross slip plane the SF between the partials must first be compressed, which requires a high stress to overcome the repulsive forces between the partial dislocations. Therefore, cross slip is suppressed and the deformation tends to be much more planar in stainless steel than in higher SF materials. These faults are precursors of the deformation twins found in the bands, since the faults and twins are formed progressively with strain. The twins can occur gradually by partial dislocation slip. The activated SFs and the subsequently initiated twin boundaries can refine the microstructure to a level similar to a nanocrystalline and UFG matrix. Yamakov *et al*.^22^ suggested that the process of deformation twinning can have a two-fold effect on the mechanical behavior of the material. First, during the early stage of plastic deformation, when the grain interiors are practically free of dislocations, it can facilitate the deformation by adding additional slip systems or by facilitating the transfer between existing slip systems through dislocation-twin reactions. Second, once twins have been nucleated they can repel certain types of gliding dislocations and give rise to pile-ups, with consequent strain hardening of the material. The importance of partial dislocations and SFs or twins in the plastic deformation of fcc metals has recently been addressed by several authors^22,27,33^. On the other hand, the twin lamellar structure may be viewed as inherently bimodal^34^ because the length scale of the twin lamellae in the two dimensions parallel to the twin boundaries (TBs) is significantly larger than the ultrafine/nano scale in the direction perpendicular to TBs. Dislocation can thus accumulate, forming tangles to subdivide the twin lamellae. Meanwhile, the TBs in large numbers also serve as the locations where a high density of dislocations can move and build up starting from low levels. Consequently, the work-hardening ability is dramatically improved in the present steel which possesses a low SFE and, especially important, NG structure.

We know that twinning and strain-induced α′- martensite are strain hardening mechanisms that restrict localization of strain and contribute to high ductility, as observed in Table 1. Twinning contributed to excellent ductility in the high strength NG/UFG structure, while strain-induced martensite governed the high ductility of CG steel. The high ductility of conventional CG austenitic steel was associated with gradual transformation of austenite to martensite that increases the strain hardening rate and delays the onset of localized necking^35–37^. In striking contrast to the behavior of CG austenite, the strain accommodation mechanism changed from transformation-induced plasticity (TRIP) in the CG alloy to twin-induced plasticity (TWIP) in the NG/UFG alloy^17^. Nanoscale twinning was a major deformation mechanism contributing to the observed excellent ductility of “high strength” NG structure, while for the “low strength” CG structure, ductility was comparably good, but due to strain-induced martensite formation at the intersection of shear bands. This was a clear case of grain size effect (and strength). The underlying reason for such a behavior was attributed to increase in the stability of austenite with decrease in grain size and explained in terms of austenite stability-strain energy relationship, such that the NG/UFG austenite resists transformation to martensite^17^.

**Figure 8:**
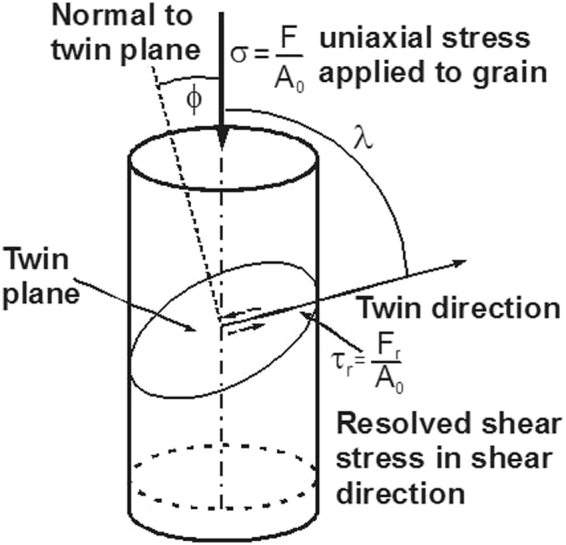
Schematic of axial compressive stress that produces a resolved shear stress leading to activation of mechanical twinning. A similar figure is applicable to represent activation of shear-bands.

The observed preference for twinning in NG/UFG steel (Fig. 3) can be explained by computing the critical shear stress (defined in Fig. 8) required to initiate twinning partial dislocation (τ_Twin_) with that required to nucleate shear-bands (τ_shear-bands_). The respective shear stresses are given by^33,38,39^:

1 $${\tau }_{{\rm{shear}}{\rm{bands}}}=\frac{2\alpha \mu {{\rm{b}}}_{{\rm{shear}}{\rm{bands}}}}{{\rm{d}}}$$

2 $${\tau }_{{\rm{Twin}}}=\frac{2\alpha \mu {{\rm{b}}}_{{\rm{Twin}}}}{{\rm{d}}}+\frac{\gamma }{{{\rm{b}}}_{{\rm{Twin}}}}$$

where μ is the shear modulus (~78 GPa) and γ is the stacking fault energy of the alloy (~30 mJ/m^2^)^10,11,20^, b_shear bands_ and b_Twin_ are the magnitude of the Burgers vector for dislocation-cell type martensite in shear-bands and Shockley partial twinning dislocations, respectively. The parameter α reflects the character of the dislocation and is ~1 for edge dislocation^33^ and contains the scaling factor between the length of the dislocation source and the grain size. At a critical grain size, the resolved shear stress τ_Twin_ and τ_shear-bands_ are equal such that by combining equations 1 and 2, the critical grain size for transition is:

3 $${{\rm{d}}}_{{\rm{C}}}=\frac{2\alpha \mu ({{\rm{b}}}_{{\rm{shear}}{\rm{bands}}}-{{\rm{b}}}_{{\rm{Twin}}})\,{{\rm{b}}}_{{\rm{Twin}}}}{\gamma }$$

**Figure 9:**
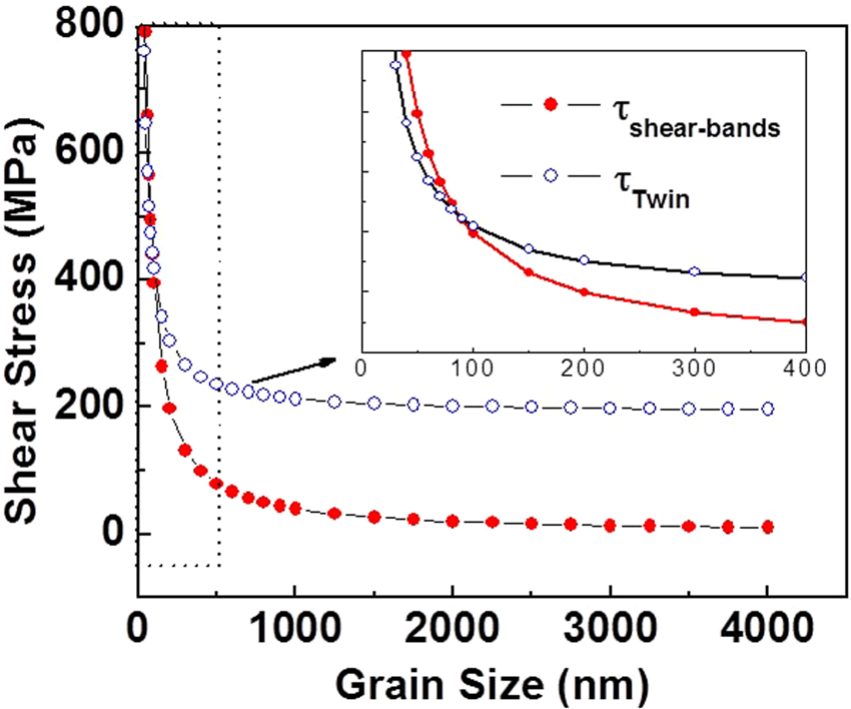
Computed shear stress (according to equations 1 and 2 as a function of grain size. For our alloy system, there is a transition in deformation mechanism at ~2100 nm grain size from twinning in NG regime to shear-bands in CG regime.

Using α = 1.5, the theoretically estimated critical grain size d_c_ is ~250 nm (Fig. 9) and the minimum stress required for twin nucleation is ~250 MPa (Taylor factor = 3.1 for fcc structure). However, experimentally twinning was observed up to SMG (~750 nm). The underlying reason for this difference is the influence of elastic anisotropy and the small Peierls-Nabarro stress that is ignored in equations 1–3. Nevertheless, equation 3 and Fig. 9 predict a transition in the deformation mechanism from twinning in NG/UFG steel to shear-bands in CG steel, consistent with the experimental observations (Figs 3–6). Thus, twinning was the deformation mechanism in the NG/UFG steel, when the critical shear stress for twinning was relatively low and strain induced martensite formation in shear-bands was the deformation mechanism in the CG steel when the critical shear stress for twinning was high (or τ_shear bands_ was low). This is in addition to the effect of austenite stability on grain size, which also impacts the deformation mechanism. It has been previously discussed that austenite stability increases with decrease in grain size^17^.

### Interplay between grain structure, deformation mechanism and fracture

There were intriguing differences in the mode of fracture (Figs 3–6) when the deformation mechanism changed from mechanical twinning in the NG/UFG structure to combination of mechanical twinning and strain-induced martensite in the FG structure, and finally to only strain-induced martensite in the CG structure, at similar level of tensile elongation. In the NG/UFG austenitic stainless steel at low magnification, the fracture surface was flat (Fig. 3b). But at high magnifications, fine striations, similar to those observed in fatigue fracture were observed, except that there was line-up of voids just along the striations. This type of fracture morphology was observed everywhere on the fracture surface and was more distinct and clear when the SEM micrographs was processed by Image Pro software. It appeared as if tearing occurred along the striations.

A similar fracture surface morphology was observed for SMG (Fig. 4b) and FG (Fig. 5b) austenitic stainless steels. In the FG austenitic stainless steel, in addition to striations, microvoid coalescence fracture, which is typically observed in ductile materials was also observed. These two-types of fracture morphology is consistent with the observed two types of deformation mechanisms, namely, mechanical twinning and strain-induced martensite. Accordingly, in the CG steel, where strain-induced martensitic transformation occurred and twinning was absent during tensile straining, microvoid coalescence was only observed. Intriguingly, the shape of microvoids observed in CG austenitic steel and to some extent in FG austenitic steel were similar to the line-up of voids just along the striations in NG/UFG and SMG steels. The microvoids corresponding to microvoid coalescence were only slightly large in size in comparison to the line-up of voids just along the striations.

**Figure 10:**
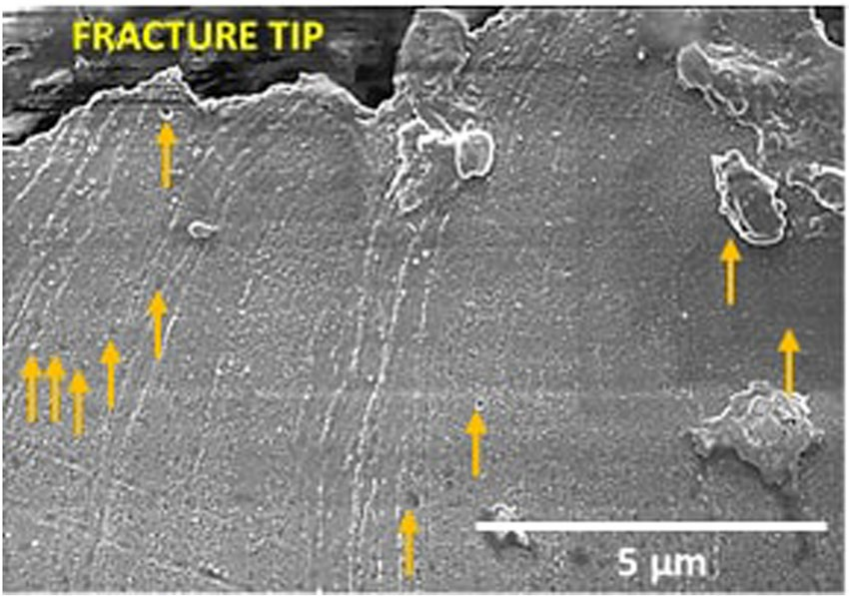
Scanning electron micrograph illustrating voids along the striations in the cross-section image of the fracture surface in NG/UFG steel. The voids are marked by arrows.

**Figure 11:**
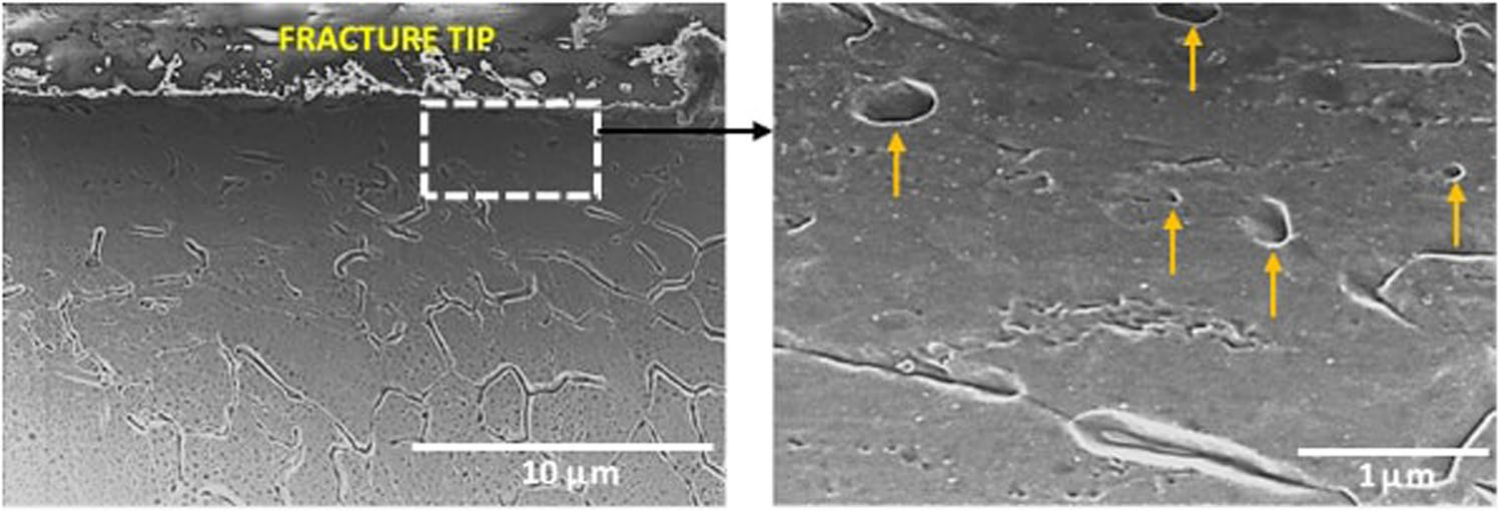
Low and high magnification scanning electron micrographs of the cross-section of the fracture surface illustrating nucleation of voids at martensitic laths in CG steel. Voids are marked by arrows.

**Figure 12:**
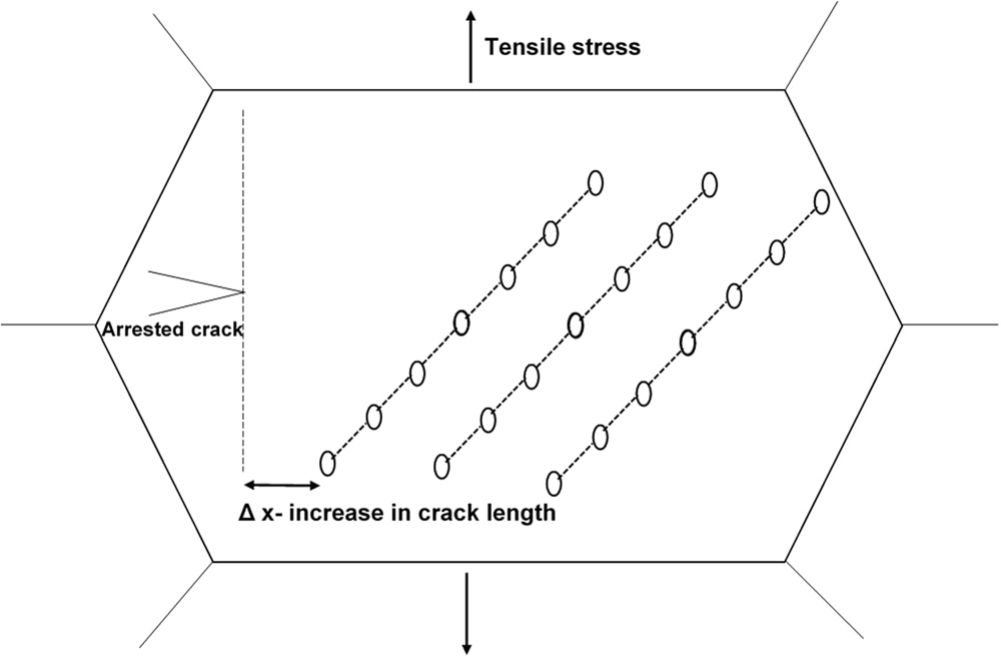
Schematic illustration of the envisaged explanation for the formation of microvoids along the striations because of straining during crack arrests (quasi-static crack growth). Δx is the increment in crack length.

To further understand the fracture process, the microstructure just beneath the fracture surface was studied via SEM. In the NG/UFG austenitic stainless steel, striations extending into the bulk of the material were observed (Fig. 10) while in the CG austenitic stainless steel, fine martensitic laths were viewed as potential sites for void nucleation (Fig. 11). The appearance of striations with spacing of ~3–8 µm suggested a step-wise (quasi-static) crack growth normal to the direction of the striations, i.e., crack propagation occurred in steps. As regards the line-up of voids along the striations, it is envisaged that the voids grow ahead of an arrested crack, and when the crack advances, the tearing of the intervoid region forms a ridge that defines the new crack front. This process repeats as a quais-static crack growth process such that multiple striations are observed. This explanation is schematically presented in Fig. 12.

## Discussion

It is clear from the aforementioned observations described in Figs 3–6 that the difference in fracture morphology between NG/UFG, SMG and CG structures is related to deformation mechanism, where FG exhibits a fracture morphology that is a combination of NG/UFG, SMG and CG structures. When mechanical twinning is the dominant deformation mechanism, striations with line-up of voids were observed. On the other hand, microvoid coalescence or dimple rupture occurred when metastable austenite transformed to martensite during tensile straining. Deformation twinning and strain-induced α′-martensite are essentially both strain hardening mechanisms that inhibit localized strain and contribute to ductility. Moreover, deformation twinning and strain-induced martensite are microstructurally similar from the viewpoint that both involve diffusionless shear of a constrained plate-shaped region in the parent crystal. The mechanism of deformation (twinning) must be related to the enhanced contribution of high fraction of grain boundaries to strength and higher stability of NG/UFG austenite that restricts strain-induced martensite^17,40^, both of which govern deformation mechanism and consequently ultimate fracture. It is widely recognized that when fcc austenite transforms to bcc martensite, it introduces anisotropic strain in the neighboring untransformed austenite. Furthermore, the near uniform distribution of transformation strain necessitates that several multi-variant transformation must simultaneously take place within an austenite grain to reduce total strain energy^2,4^. But when the austenite grains are smaller than the martensite lath, as in the NG/UFG structure, the possibility that number of variants of martensite to simultaneously participate within an austenite grain is appreciably reduced because of very high strain energy (~850 MJ/m^3^), which reduces the ability to potentially nucleate martensite. Thus, a single variant is favored or preferred for strain-induced martensitic transformation to occur in the NG/UFG structure^41^. But this requires a significant reduction in the strain energy for martensitic transformation to take place in the NG/UFG austenite and is not possible because the elastic strain energy is very high (~850 MJ/m^3^)^41^. The insights on the relationship between grain size and plasticity (inclusive of fracture) mechanisms are important in providing directions for the futuristic science-based design of high strength-high ductility combination bulk NG/UFG materials. There are no reports of studies that show a systematic study of grain size and deformation behavior to the fracture mechanism in relatively large grain size spectrum from (NG/UFG to CG regime) in “a single material” using an “identical processing approach”. To develop an unambiguous understanding, it is desirable to produce structures with systematically varying grain size in a single material processed by a single set of parameters. The discovery of the “grain size-deformation behavior” and relationship to “fracture mechanism” in the study described here is fundamentally important for designing material structures with optimal mechanical properties.

## Conclusions

The study provided insights on grain size-deformation mechanism-fracture relationship in austenitic stainless steels from NG/UFG to CG regime. In NG/UFG and SMG structure, when twinning was the mechanism of deformation, the fracture morphology was characterized by striations (river markings) with line-up of voids just beneath the striations. In contrast, in the CG structure, microvoid coalescence type of fracture occurred. A distinct transition from striated fracture with line-up of voids to microvoid coalescence occurred in FG steel, consistent with transition of deformation mechanism from twinning to strain-induced martensite. The appearance of striations suggested a stepwise (quasi-static) crack growth normal to the direction of striations. The voids grew ahead of an arrested crack, and when the crack advanced, the tearing intracavity (or intervoid) formed the ridge that defined the new crack front.
